# Field Evaluation of Chemotherapy on HLB-Affected Citrus Trees With Emphasis on Fruit Yield and Quality

**DOI:** 10.3389/fpls.2021.611287

**Published:** 2021-02-22

**Authors:** Muqing Zhang, Palaniyandi Karuppaiya, Desen Zheng, Xiuxiu Sun, Jinhe Bai, Rhuanito S. Ferrarezi, Charles A. Powell, Yongping Duan

**Affiliations:** ^1^State Key Laboratory for Conservation and Utilization of Subtropical Agro-Bioresources, Guangxi University, Nanning, China; ^2^Indian River Research and Education Center, University of Florida, Fort Pierce, FL, United States; ^3^US Horticultural Research Laboratory, USDA-ARS, Fort Pierce, FL, United States

**Keywords:** Huanglongbing, Liberibacter, flavor, nutrition, chemotherapy

## Abstract

Huanglongbing (HLB) is one of the most devastating diseases of citrus, which is associated with *Candidatus* Liberibacter asiaticus (Las) in the United States. To date, no effective antimicrobial compound is commercially available to control the disease. In this study, we investigated the effects of different antimicrobial chemicals with suitable surfactants on HLB-affected matured citrus trees with emphasis on the fruit yield and quality. Each treatment was applied three times in a 2-week interval during the spring flush period, one time in summer and three times during the autumn flushing period. We extensively examined different parameters such as pathogenic index, disease index, tree canopy, fruit yield, quality, and nutritional status. The results showed that among the treatments, penicillin (PEN) with surfactant was most effective in suppressing Las titer in infected citrus trees, followed by Fosetyl-Al (ALI), Carvacrol (CARV), and Validamycin (VA). Fruit quality analysis revealed that PEN treatment increased the soluble solids content (SSC), whereas Oxytetracycline (OXY) treatment significantly reduced titratable acidity (TA) level and increased the SSC/TA ratio compared to the control. Nutrient analysis showed increased N and Zn levels in ALI and PEN treatments, and OXY treatment increased leaf P, K, S, and Mg levels compared to untreated control. Furthermore, B, Ca, Cu, Fe, and Mn in leaves were reduced in all chemical treatments than that of the untreated control. These findings revealed that some of the chemical treatments were able to suppress Las pathogen, enhance nutritional status in leaves, and improve tree growth and fruit quality of HLB-affected trees.

## Introduction

Citrus is one of the largest horticultural crops around the world, with great economic, high nutrition, and health benefits (Calvaruso et al., 2020). Orange juice is the most consumed fruit juice worldwide for its flavor and nutritional values (Baldwin et al., 2017; Dala-Paula et al., 2019). In the United States, orange and grapefruit for the juice market are produced in Florida, while citrus fruits for consumption as fresh fruit are grown mainly in California, Arizona, and Texas. In terms of production, Brazil is the largest orange producer in the world. The global production of orange juice is projected to fall by 23% to 1.6 million tons by 2019/2020, as production is forecasted to decrease in Brazil and Mexico.

It is estimated that global orange fruit production for 2019/20 will fall by 7.8 million metric tons from the previous year to 46.1 million (USDA-FAS, 2020). The Florida citrus industry is still fighting for its survival since millions of infected trees have entered a severe decline with premature fruit drops and diebacks. Hence, an orange box’s price had increased 3.2 times from $2.89 to $9.34 since Huanglongbing (HLB) was detected in the United States (Singerman et al., 2017). Despite considerable research efforts, there is no cure for the disease, and orange production in Florida dropped from around 244 million boxes in the 1997–1998 season to 67.65 million boxes in the 2019–2020 season (USDA-NASS, 2020).

Citrus is vulnerable to a large number of diseases caused by bacteria, fungi, nematodes, parasitic, viruses, viroids, and other graft-transmissible pathogens. Among them, HLB, this century-old disease, has become the most devastating disease worldwide. Huanglongbing is a phloem-limited bacterial disease present worldwide except in Australia, the Pacific Ocean, Western Asia, and the Mediterranean basin (Massenti et al., 2016). The causal agents of HLB are associated with three species of *Candidatus* Liberibacter that are vectored at least by two psyllid species, *Diaphorina citri* in Asia and America, and *Trioza erytreae* in Africa. In many countries, citrus growers are encountering significant challenges with the outbreak of HLB, especially the one caused by *Ca.* Liberibacter asiaticus (Las) (Dala-Paula et al., 2019). HLB-affected plants systemically display a range of symptoms, including the changes of physiological, biochemical, and fruit-sensory characteristics (Baldwin et al., 2018; Dala-Paula et al., 2018). When the *Liberibacter* bacterium is transmitted into the citrus plant, it causes a cascade of events in infected plants, including phloem dysfunction, callose deposition, and carbohydrate over-accumulation in leaves, which leads to the restriction of nutrient uptake, transport, and reduction of photosynthesis (Fan et al., 2010).

Huanglongbing also affects the yield and quality of citrus fruits by causing abnormal coloration and reduction of size and shape. These symptomatic fruits are likely to have lower sugars, higher acids, bitter limonoids and astringent flavonoids, particularly in those harvested in earlier season (Baldwin et al., 2010). Flavors of the HLB affected oranges are often altered by having lower sugars, but higher limonoids and flavonoids (Plotto et al., 2010). Previous studies have demonstrated that antibiotics treatments not only reduce Las titers but also increase fruit yield and quality (Halbert and Manjunath, 2004; Shokrollah et al., 2011; Zhang et al., 2012, 2014; Hussain et al., 2019). Chemical treatments, such as penicillium (PEN), combined with heat and nutrition treatments as an integrated strategy is effective against Las, and enhances new growth of canopy of HLB-affected trees (Zhang et al., 2019). Unfortunately, there is no resistant cultivar or other commercially available cure though intensive research and progresses have been made toward the finding of effective solutions for HLB.

To continue our research efforts on chemotherapy (Zhang et al., 2019), we emphasized this study on fruit yield and quality, aiming to mitigate HLB-induced off-flavor in the fruit and juice quality, and to improve nutritional values in leaves of HLB-affected trees by using chemotherapy treatments.

## Materials and Methods

### Experimental Field

The experiment was carried out on a randomized block design in a citrus grove located in Fort Pierce, FL, United States. Citrus trees with typical HLB symptoms were identified and tagged for the chemotherapy treatments.

### Plant Material and Cultural Practices

Three-year-old “Valencia” sweet orange (*Citrus sinensis* [L] Osbeck) trees grafted on Kuharske citrange [*C. sinensis* (L.) Osbeck × *Poncirus trifoliata* (L.) Raf.] rootstock were used on this trial. Trees were planted September 2013 in high-density staggered planting with two double-row configurations with fertigation and micro-sprinkler irrigation (Ferrarezi et al., 2020). Each row consisted of two staggered sub-rows spaced 1.5 m apart with 2.7 m between trees, and spaced 6.1 m apart from the other row in the bed (total of 955 trees per ha). The amount of nitrogen (N) applied to trees varied by tree age and was based on UF/IFAS recommendations (Morgan and Kadyampakeni, 2020). During non-bearing years, each tree received 0.11 kg (2013/2014), 0.20 kg (2014/15), and 0.34 kg (2015/16) on an annual basis. During fruit-bearing years (2016/17 to 2019/20), we applied 224 kg N ha^–1^. Fertigation was used to deliver nutrients to all trees. Water-soluble fertilizer containing 15-11-31 (Agrolution pH Low; ICL, Summerville, SC) was applied weekly. The 15 % N component consisted of nitrate-N (8.5 %), ammonium-N (0.9 %), and urea-N (5.6 %). The phosphorus (P) component was derived from ammonium phosphate, monopotassium phosphate, and phosphoric acid. In addition to monopotassium phosphate, potassium nitrate was used to supply potassium (K). This blend also contained 0.8% Mg, 1.0% S, 0.02% B, 0.05% Cu, 0.10% Fe, 0.05% Mn, 0.0005% Mo, and 0.05% Zn. These nutrients were derived from MgSO_4_, boric acid, EDTA of Cu, Fe, Mn, and Zn, and (NH_4_)_2_MoO_4_. For irrigation, one microsprinkler emitter (Fan-Jet PLUS; Bowsmith, Exeter, CA) with an output of 64 L h^–1^ at 138 kPa, a 4.8 m diameter wetted pattern, and an irrigation efficiency of 91 % measured in 2017 by the FDACS Mobile Irrigation Labs (2020) was installed every two trees. Irrigation management followed recommendations by Morgan and Kadyampakeni (2020) for non-bearing (years 2013–2016) and bearing trees (>2017). Reference evapotranspiration was monitored from 2013 to 2017 using a local weather station (Mini-Weather Station; Hunter Industries, San Marcos, CA) and from 2017 to 2019 by using the Florida Automated Weather Network (FAWN) station in St. Lucie West, FL. Scouting for pests and diseases occurred monthly, and control measures were implemented in accordance with the UF/IFAS best management practices (Diepenbrock et al., 2019).

### Chemotherapy Treatment

Five chemical treatments were applied by foliar spraying, and tap water (CK) was used as a control treatment. Every treatment was replicated three times, and each replication was formed by 10 trees. Foliar spray treatments were applied three times at fortnight intervals during the spring flush season, one in the summer flush period, and three times during the fall flush season. The foliar spraying method employed a 100-gallon sprayer with an adjustable nozzle and 55 psi gun (GNC Industries INC, AR, United States). For each treatment, trees were sprayed with the appropriate chemical concentration to runoff. The treatments and chemical concentrations required for each chemical treatment with surfactant were as follows: **PEN:** Penicillin G potassium salt at 5000 ppm with Siltrate silicone surfactant (Positive control); **ALI:** Aliette^®^ WDG Aluminum tris (O-ethyl phosphonate) at 5 lbs/acre rate with Spread-R 90/10 non-ionic surfactant (Bayer CropScience LP. EPA Reg. No. 264–516); **OXY:** Mycoshield^®^ Oxytetracycline calcium complex at 1.5 lbs/acre rate with balance pH acidifier/surfactant (NuFarm Americas, Inc. EPA Reg. No. 55146-97); **CARV:** Carvacrol essential oil at 1000 ppm with Siltrate silicone surfactant/penetrant (Tokyo Products International America. CAS No. 499-75-2); **VA:** Validamycin antimicrobial at 500 ppm with Siltrate silicone surfactant/penetrant (Qianjiang Biochem, China); and **CK or Control:** Distilled Water as Control (Negative control).

### DNA Isolation, Las Bacterial Titer, and Pathogen Index

Citrus tree branches with typical HLB symptoms were identified to quantify Las bacterial titers. Mature leaf samples from the symptomatic branch of HLB were taken from April 2015 to December 2017 at 4-month intervals. The symptomatic leaf samples collected were kept in an icebox to avoid direct sunlight and kept in −80 °C until DNA isolation. DNA isolation was performed using the CTAB method for the quantification of the Las bacterial titer.

A total of 180 symptomatic leaf samples collected from citrus trees in each grove were screened for Las bacterial titer using Real-time Polymerase Chain Reaction (RT-PCR) analysis. The qRT-PCR analysis was performed using Las-specific primers and probes, as described in Li et al. (2006). Based on the Cycle threshold (Ct) value, the bacterial titers were assigned as follows: category 0 = Ct ≥ 36.0; category 1 = 32.0 ≤ Ct < 36.0; category 2 = 28.0 ≤ Ct < 32.0; category 3 = 24.0 ≤ Ct < 28.0 and category 4 = Ct < 24.0. The pathogenic index (PI) were calculated for each treatment as described in (Yang et al., 2016).

### Citrus Tree Growth, Scoring, and Disease Index

Citrus tree growth was measured in July 2017, February 2018, and December 2018. Eight trees were selected randomly in each treatment excluding border trees. Trunk diameter was measured at 8 cm above the graft union in a north-south direction using a digital caliper. Tree height was measured from the soil surface perpendicular to the tallest branch using a meter stick (Model No. 98024; Seco Manufacturing, Mound City, IL). Canopy width or diameter was measured parallel (north-south direction) and perpendicular (east-west direction) to the tree row. The canopy volume was estimated according to the formula as follows (Bowman et al., 2016):

$$\mathrm{Canopy}\,\mathrm{volume}=\frac{{\text{width}}^{2}\times \text{height}}{4}$$

Tree health was evaluated using scores based on the observable amount of HLB decline, also known as disease indexing (DI). The same skilled field manager scored all 180 treated trees at 12-month intervals over three years using a 0–4 scale, where category 0 = No HLB symptoms, normal fruit load, and size, normal leaf size and growth flushes; category 1 = Some HLB symptoms, modest fruit load and normal fruit size, mostly normal growth flushes; category 2 = Some HLB symptoms, modest fruit load with some smaller fruit sizes, most growth normal; category 3 = Obvious HLB symptoms, light fruit load and multiple small fruit sizes, modest or no growth flushes; category 4 = Obvious HLB symptoms, including small leaves and dead wood, small fruit size and virtually no new growth^1^.

The disease index (DI) was calculated for each treatment as follows:

$$D\,I=\,\sum_{n=0}^{4}\frac{S\,u\,m\,o\,f\,a\,l\,l\,n\,u\,m\,e\,r\,i\,c\,a\,l\,s\,c\,o\,r\,e\,s}{\mathrm{Total \#}\,\mathrm{plants}\,\mathrm{counted}\times \mathrm{maximum}\,\mathrm{score}}\times 100$$

### Citrus Fruit Number, Size and Yield

For determination of fruit number and size, fruit from eight trees per plot, excluding border trees, were sorted by an optical sizer (Autoline; Reedley, CA) following the method described by Phuyal et al. (2020). Tiny, diseased, and damaged fruit were discarded. Fruit size was used to calculate fruit diameter as follows: (average diameter in each fruit count category number of fruits in that category) ÷ total fruit count. To determine fruit yield, fruit from each individual tree per plot was handpicked and weighed using a portable scale (D51P60HR1; Ohaus, Los Angeles, CA). Total fruit yield per hectare (Ha) was calculated by extrapolating fruit yield per tree to area basis.

### Citrus Fruit Quality

For fruit quality determination, 20 uniform-sized fruit ranging from 90 to 95 mm diameter were selected. Fruit were weighed and passed through a juice press (model 2720; Brown International Corporation, Covina, CA). Juice volume and juice weight were recorded. Soluble solids content (SSC) of the juice was measured using a refractometer (HI96801; Hanna Instruments, Woonsocket, RI). Fruit titratable acidity (TA) was measured with an automatic potentiometric titrator (HI931; Hanna Instruments, Woonsocket, RI). For titration, 25 mL of sample juice was diluted with deionized water to make 50 mL. The solution was later titrated using a 1N sodium hydroxide solution. Fruit quality was also expressed as the ratio of soluble solids content and titratable acidity. Juice yield was calculated and expressed as yield of solids per ha by the equation: [(% of juice in fruit÷100) × (soluble solids content÷100) × fruit yield] (Rewal and Jhooty, 1985). The SSC/TA ratio was calculated by dividing the SSC by TA obtained.

### Leaf Nutrient Analysis

To investigate the leaf nutrients, 4- to 6-month-old fully expanded leaves from non-fruiting terminals were randomly chosen from each chemical treatment replication (Morgan and Kadyampakeni, 2020). The leaves were washed with tap water to clean the dust, followed by distilled water. Then the leaves were dried in the hot air oven at 80°C for 48 h. The dried samples from each plot were ground to pass a 1-mm mesh screen (Wiley Laboratory Mill Model 4 3375-E10; Thomas Scientific, Swedesboro, NJ). Five grams of leaf sample were analyzed using the dry-ashing method and assessed by inductively coupled plasma atomic emission spectroscopy to determine the concentration of P, K, calcium (Ca), Magnesium (Mg), S, boron (B), copper (Cu), Fe, Mn, and Zn. Leaf N concentration was measured by macro dry combustion using an elemental analyzer (LECO CNS-2000; LECO Corporation, St. Joseph, MI). All the procedures of digestion, distillation, and titration were carried out as described (Bremner, 1965).

### Statistical Data Analysis

To evaluate the effect of each antimicrobial, pathogenic index (PI) was used to calculate the efficacy of each treatment (Zhang et al., 2019). To avoid the treatment’s background effects, the relative efficacy (E_r_) for each treatment was determined according to the following equation adopted by Rewal and Jhooty (1985):

$$\text{Er}=\frac{P\,I_{0}\times 1+{\text{Δ}}_{c\,k}-P\,I_{t}}{P\,I_{0}\times 1+{\text{Δ}}_{c\,k}}\times 100$$

Where PI_0_ = PI at pretreatment; Δ_ck_ = the control increment of PI; PI_t_ = PI at 1 year after initial treatment.

The control increment of the PI (Δ_ck_) was calculated by:

$${\text{Δ}}_{c\,k}=\frac{{\text{PI}}_{0}-{\text{PI}}_{T}}{{\text{PI}}_{0}}\times 100$$

Where PI_0_ = PI of control at pretreatment; PI_T_ = PI of control at post-treatment.

All the experiments were repeated in triplicate with ten infected trees each. The slope for each chemical treatment was determined (Zhang et al., 2019). The control and each chemical treatment were analyzed using Repeated Measures one-way ANOVA test, followed by Dunnett’s multiple comparisons.

Data were analyzed by year for two consecutive seasons (2017–18 and 2018–19) using SAS 9.4 (SAS Institute, Cary, NC). A generalized linear mixed model was used to analyze error variance, with treatments entered as fixed effects and block as a random effect. The data were checked for assumptions of the linear model. Log transformation and square root transformation was executed as needed. Data were subjected to analysis of variance by the F test and, when significant, multiple comparisons were assessed by Tukey’s *post hoc* honest significant difference test (*P* ≤ 0.05).

## Results

### Chemical Treatments Reduced Las Bacterial Titers in Infected Plants

**FIGURE 1:**
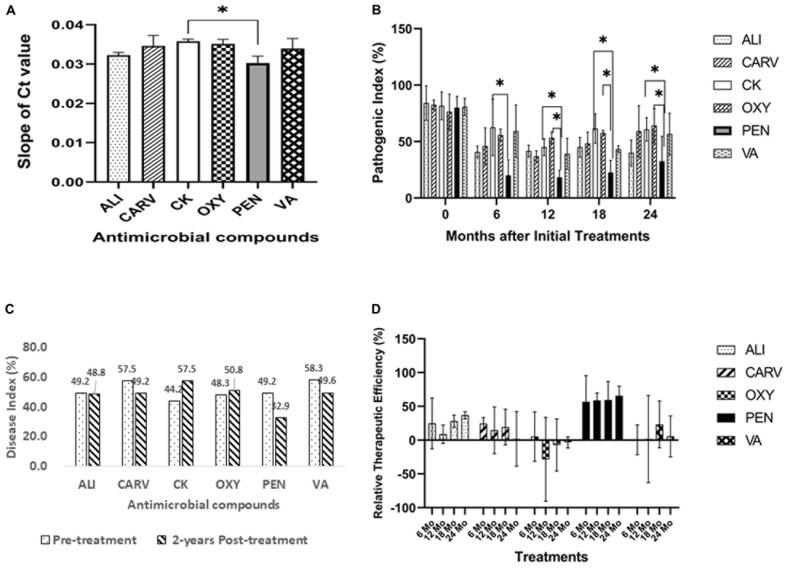
Effect of chemotherapy on HLB-affected citrus trees. **(A)** Slope of Ct values. **(B)** Pathogenic Index. **(C)** Disease Index. **(D)** Relative therapeutic efficiency. Statistically significant differences between the chemical treatment and control were indicated by ^∗^*P* < 0.05.

Las bacterial titers were detected in typical HLB symptomatic citrus trees, and Ct values ranged from 17 to 32. The slope value of PEN treatment was significantly reduced compared to control, followed by ALI, VA, OXY, and CARV treatment (Figure 1A). A pathogen index was calculated for each chemical treatment bi-annually for 2 years to lower the trees ambient effect (Figure 1B). At each bi-annual period, the pathogen index significantly differed between chemical treatments. The highest Ct values and lowest pathogen index were observed in the trees treated with PEN throughout the test periods, followed by ALI treatment. The lowest disease index was obtained at regular intervals over 2 years after initial therapy from PEN-treated citrus trees, followed by ALI (Figure 1C). Compared to the untreated control, trees treated with CARV, OXY, and VA exhibited no significant discrepancies in Las bacterial titer and disease index.

The relative therapeutic efficacies were calculated from pathogen index for each treatment (Figure 1D). The average therapeutic efficacy from pathogen index was 16.1% in the first year (2017) after the initial application, then increased up to 28.0% in 2018 (Year 2) and 17.8% in 2019 (Year 3). PEN was most effective with the highest therapeutic efficiency, followed by ALI and CARV. OXY was not effective in this study.

### Chemical Treatments Increased Canopy Height, Volume, Trunk, and Yield of HLB-Affected Trees

**FIGURE 2:**
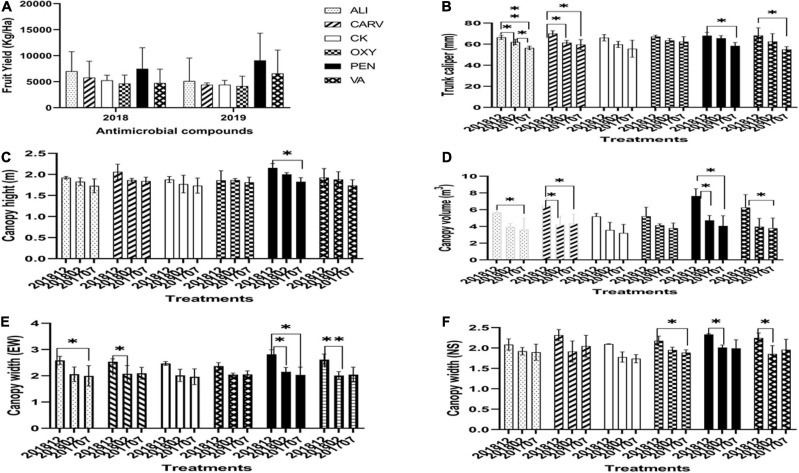
Citrus growth and yield in response to chemical treatment. **(A)** Fruit yield. **(B)** Trunk caliper. **(C)** Canopy height. **(D)** Canopy volume. **(E)** Canopy width (EW). **(F)** Canopy width (NS). Statistically significant differences between the chemical treatment and control were indicated by ^∗^*P* < 0.05 and ^∗∗^*P* < 0.01.

Two-year-fruit yields showed that PEN treatment produced the maximum fruit yield (over 8000 Kg/Ha) compared to control followed by VA, and ALI, while the lowest yield was recorded in OXY treatment (Figure 2A). The citrus tree trunk’s caliper increased over time and was higher for treatment with CARV, PEN, VA, and ALI (Figure 2B). The maximum tree canopy height was recorded 2 years after PEN application (Figure 2C). In canopy volume and width (East to West) analysis, PEN treatment was significantly increased after 2 years of initial treatment, followed by CARV, ALI, and VA (Figures 2D,E) compared to control. On the other hand, the canopy width (North to South) measurement study showed no significant difference as compared to control (Figure 2F).

### Fruit Quality Increased in Some Chemical Treatments

**FIGURE 3:**
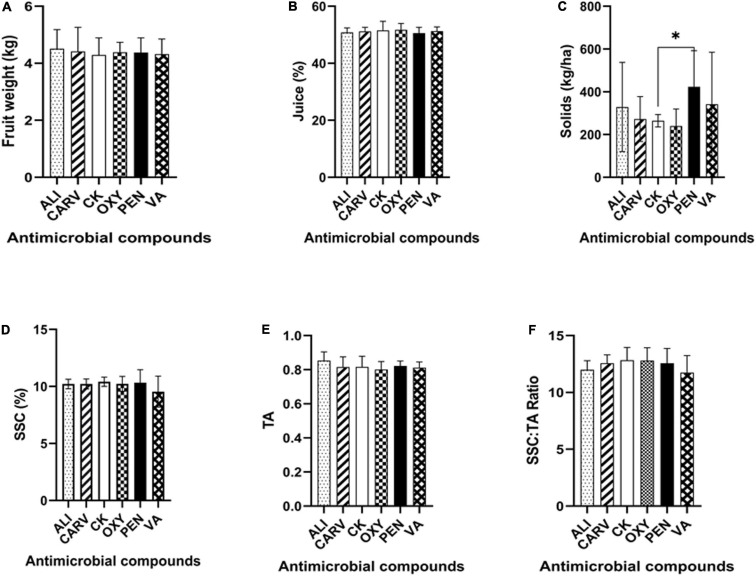
Analysis of citrus fruit quality under each chemical treatment. **(A)** Fruit weight. **(B)** Juice percentage. **(C)** Solids. **(D)** Soluble sugar content. **(E)** Titratable acidity. **(F)** Soluble sugar content/Titratable acidity ratio (SSC/TA). Statistically significant differences between the chemical treatment and control were indicated by ^∗^*P* < 0.05.

The maximum fruit weight was obtained in ALI treatment in comparison to the control (Figure 3A). The percentage of juice was increased in all treatments as comparable to control. The findings of this analysis revealed that there is no significant difference in the percentage of juice among the treatments tested (Figure 3B). The therapy with PEN exhibited significantly higher solids than control, followed by VA, ALI, and CARV, while OXY recorded the lowest solids (Figure 3C). Figure 3 also summarized the mean and standard deviation of SSC in each chemical treated HLB citrus tree fruits. This study found no significant difference in SSC in all chemical treatments, while trees treated with VA showed the lowest SSC percentage (Figure 3D). The highest TA was recorded in control, while the lowest TA was noted in OXY treatment (Figure 3E). The highest SSC/TA ratio was found in untreated control, while the lowest ratio was recorded in VA treatment (Figure 3F).

### Chemical Treatments Increased Nutritional Content of Leaves

In the present study, macro- and micro-nutrients such as N, P, K, Ca, Mg, S, B, Cu, Fe, Mn, and Zn were examined in the HLB-affected leaves treated with various chemical treatments. Our results found that the N content was significantly increased in ALI treatment in comparison to untreated control, while the leaf N content was significantly reduced in CARV treatment (Table 1). The concentration of P, S, Mg, and Zn was not considerably affected by the chemical treatments. Compared to control, the concentration of B, Cu, Fe, Ca, and Mn was reduced in HLB-affected leaves under all chemical therapies (Table 1). Interestingly, leaf K concentration increased in all the chemical treatments except the control (Table 1).

**TABLE 1:** Leaf nutrition status of citrus trees treated by antimicrobial compounds.

**Leaf nutrition**	**ALI**	**CARV**	**CK**	**OXY**	**PEN**	**VA**
N (%)	2.900.168a	2.430.061b	2.560.13b	2.700.042ab	2.610.153ab	2.600.105ab
P (%)	0.1830.006a	0.1800.01a	0.1770.006a	0.2000.043a	0.1770.025a	0.1900.04a
K (%)	1.7130.051a	1.6570.254a	1.4470.040a	1.6770.315a	1.6070.065a	1.6170.384a
Ca (%)	3.880.200a	4.1270.435a	4.4730.140a	4.1230.217a	3.810.358a	3.7870.145a
Mg (%)	0.3070.015a	0.30.01a	0.2830.011a	0.3370.060a	0.30.03a	0.3130.065a
S (%)	0.3130.006a	0.320.026a	0.3330.006a	0.3470.012a	0.3030.047a	0.3130.021a
B (ppm)	47.075.213a	49.866.459a	59.193.114a	53.613.995a	44.657.153a	49.467.418a
Fe (ppm)	75.757.96a	81.2424.24a	103.472.30a	85.3016.80a	71.7115.85a	76.489.76a
Zn (ppm)	16.271.03a	15.794.01a	15.933.22a	15.052.92a	20.276.38a	16.221.10a
Cu (ppm)	72.6524.91a	101.6832.27a	120.0419.92a	94.8149.14a	77.347.57a	62.3624.18a
Mn (ppm)	8.960.24a	8.920.35a	13.443.69a	9.931.326a	9.541.797a	12.112.56a

*^*a*,*b*^Means followed by same letter were not significantly different at 0.05 level.*

## Discussion

Huanglongbing has devastated the Florida’s citrus industry by causing dramatic reduction of growing acreages and poor yield and quality of citrus production per acre. Growers depend on the discovery of the appropriate antimicrobial agent to completely eliminate/suppress Las to restore growth and productivity of infected trees. Our current study has investigated various antimicrobial chemicals with appropriate surfactant/penetrant in infected citrus trees to restore citrus tree growth, fruit production and fruit quality for over 2 years. The slope of Ct values, pathogen index and disease index showed effectiveness of each chemical treatment in controlling HLB pathogen. The results also revealed different effects on Las bacterial titers among these chemical treatments. This variation of bacterial titers in each treatment may be attributed to various factors, such as the severity of disease in treated trees and environmental condition that favors HLB pathogen (Hu et al., 2018; Zhang et al., 2019).

Chemical treatments, including PEN, ALI, and CARV, showed their efficacy in eliminating Las bacteria in citrus trees over the 2 years. PEN, a beta-lactam antibiotic, binds to penicillin-binding proteins (PBPs) and inhibit the formation of peptidoglycan cross-links in the bacterial cell wall, promoting plant growth and development (Zhang et al., 2011). In a previous 3-year field trial, PEN treatment was effective in suppressing HLB pathogen in citrus groves followed by ALI treatment (Zhang et al., 2019). The present study also indicated that the PEN was the most effective treatment for suppressing Las pathogen in infected citrus trees. ALI induces natural disease resistance in agricultural and horticultural crops (Fenn and Coffey, 1987). In a 3-year field trial, ALI treatment significantly reduced the HLB pathogen index from 64 to 44.8% (Zhang et al., 2019). In line with the previous study, ALI with non-ionic surfactant treatment effectively suppressed the Las bacterial titers over 2-year after initial treatment. CARV is a monoterpenoid phenol that can inhibit bacterial growth (Memar et al., 2017). In this study, CARV combined with silicone surfactant also showed efficacy in killing Las in the first-year, and as time progress effectiveness declined. Stansly et al. (2014) pointed out that the Ct values of Las showed discrepancies over time in each treatment and decreased Ct values from November 2008 to October 2011. Like previous studies, we also found that Las bacterial titers varied over time for each treatment, but usually decreased from 12 to 24 months in the field trial. In addition, we also analyzed the antimicrobial residues (Supplementary Table 1). The residual concentrations in the juice were below the detection limit of the instrument (5 ppb for penicillin, 2 ppb for carvacrol and 0.2 ppm for Aliette), suggesting that these residuals were below the maximum residue level for the European Union and the U.S. Citrus. The oxytetracycline concentration in the juice was 95.71 ± 9.16 ppb, which was below the maximum residue level (100 ppb) for the Joint FAO/WHO Expert Committee on Food Additives (JECFA).

Huanglongbing causes premature fruit drop, reduction of fruit size, and sometimes abnormal coloration, which reduces the yield of citrus fruit from 30% to 100% (Bassanezi et al., 2011; Dala-Paula et al., 2019). In addition, many studies reported that juice content and weight of symptomatic fruits were significantly decreased as compared to asymptomatic and/or healthy orange fruits (Bassanezi et al., 2011; Liao and Burns, 2012; Massenti et al., 2016). This study revealed a positive impact of ALI and PEN treatments on fruit weight, yield, and juice content, respectively, in a 3-year trial of matured HLB-affected trees. Similarly, Shokrollah et al. (2011) also reported the highest percentage of pulp, juice, and fruit weight when antibiotics combined with gibberellic acid and foliar fertilizer were applied to HLB-affected citrus trees.

Bowman et al. (2016) pointed out that canopy height, diameter, and volume were significantly reduced in severely HLB-affected “Valencia” trees. However, canopy volume and trunk diameter were increased in imidacloprid treated HLB-affected citrus rootstock/scion than untreated control (Stover et al., 2016). Similarly, this study also found that all chemical treatments exhibited positive effects overtime on HLB-affected trees by restoring the volume, width, and citrus tree canopy height.

The physical and chemical properties of citrus fruits are instrumental in determining juice quality. To examine HLB’s impact, factors that may affect chemical and physical properties of orange juice, such as harvest date, maturity, and pulp content should be considered (Dala-Paula et al., 2019). Several research reports demonstrated that variations due to harvest time are apparent rather than differences due to HLB (Baldwin et al., 2010; Bassanezi et al., 2011; Slisz et al., 2012; Kiefl et al., 2017; Dala-Paula et al., 2018). In this study, maximum SSC were obtained in PEN treatment as compared to control. A previous study indicated that the highest SSC recorded in Ampicillin sodium combined with Rifampicin treatment (11%), whereas lower SSC was found in control (8.83%) (Hussain et al., 2019).

Soluble solids content, the main component of citrus flavor, is a critical feature used for determining the quality of citrus fruits. The SSC is also a crucial parameter for guiding management measures and determining a picking period. The internal quality of fruit harvested in a time point may vary due to the individual difference of biological characteristics, cultivation environment, and management (Li et al., 2020). Earlier studies indicated that there are discrepancies and similarity in SSC percentage between healthy and HLB symptomatic fruit juices (Raithore et al., 2015; Massenti et al., 2016; Baldwin et al., 2018; Dala-Paula et al., 2018). Our study found that the SSC of fruits from all the chemical treatments showed no significant difference than that of control, whereas VA treatment showed the lowest SSC in the fruit juice.

The determination of fruit acidity is based on TA and/or pH, which is a crucial factor influencing the fruit (Etienne et al., 2013). HLB-affected orange juice pH was either higher, lower, or similar to the natural orange juice (Plotto et al., 2008, 2010, 2017). The highest TA was recorded in control (2.12%), while the lowest TA was observed in Ampicillin sodium in conjunction with Rifampicin (0.72%) (Hussain et al., 2019). Like the previous study, we also found the lowest TA in treatment with OXY in infected fruit juice compared to untreated control. Several studies demonstrated that TA levels were significantly increased in symptomatic and asymptomatic HLB-affected “Hamlin” orange juice than that of healthy orange juices (Plotto et al., 2010; Liao and Burns, 2012; Baldwin et al., 2018; Hung and Wang, 2018). On the contrary, there was no significant difference in TA levels in “Hamlin” orange juices obtained from HLB-affected as compared to healthy fruit juices (Raithore et al., 2015). Interestingly, Baldwin et al. (2010) revealed that asymptomatic and healthy fruit juices showed no significant difference in TA levels during December 2007, whereas decreased TA levels were noticed in asymptomatic “Hamlin” fruit juice compared to healthy in February 2008.

The SSC/TA ratio, a standard fruit quality index (ripening index), generally increases in the later harvest phase and is affected adversely by harvesting time and citrus varieties (Baldwin et al., 2010). When taking SSC/TA ratio into account, fruits of high and medium fruit load treatments were found to mature 1.5 months longer than control fruits. The SSC/TA ratio of fruits of all treatments was about 13.6 at maturity (Poiroux-Gonord et al., 2012). Several studies found discrepancies in SSC/TA ratio with asymptomatic HLB and healthy orange juices in “Hamlin” and “Valencia” sweet orange (Baldwin et al., 2010; Dagulo et al., 2010; Massenti et al., 2016; Hung and Wang, 2018; Dala-Paula et al., 2019). A recent study reported that TA, SSC, and SSC/TA levels of HLB-affected citrus fruit juice (asymptomatic) and healthy oranges exhibited a similarity pattern (Dala-Paula et al., 2019). Bai et al. (2016) suggested that healthy orange becomes more orange in color, the juice content decreases, the SSC increases, and the TA and citric acid decreases during the maturation season. Mandarin orange trees treated with OXY in conjunction with GA_3_ and foliar fertilizer had the lowest TA and the highest SSC and SSC/TA ratios (Shokrollah et al., 2011). Consistent with previous investigations, we also noticed the highest SSC/TA ratio was obtained in OXY treatment, followed by PEN.

The leaf symptoms of HLB are easily confused with nutrient deficiencies. Consequently, many investigations were carried out to understand the relationship between nutrition and HLB symptoms (Razi et al., 2011; Gottwald et al., 2012; Nwugo et al., 2013; Shen et al., 2013; Zhao et al., 2013). Mineral nutrients are pivotal in plant disease control, either promoting plant defense systems or suppressing pathogen progression (Handique et al., 2012). A study reported that leaf N, P, Ca, Mg, S, B, Cu, Fe, Mn, and Zn decreased in HLB-affected trees compared to asymptomatic and healthy trees. On the other hand, leaf K significantly increased in HLB-affected “Valencia” and “Hamlin” citrus trees. In contrast, K decreased in leaves from asymptomatic HLB-affected trees. However, the increasing in K concentration appeared only in HLB-affected leaves (Spann and Schumann, 2009). This study showed significantly higher N and Zn levels in ALI and PEN treatment, while OXY treatment increased concentrations of P, K, S, and Mg compared to untreated control. However, B, Ca, Cu, Fe, and Mn in leaves were reduced in all chemical treatments in comparison to the untreated control. In HLB-affected pre-symptomatic and symptomatic plants, K concentration was increased by 12% and 21%, respectively, compared to control plants (Nwugo et al., 2013). Disrupting the K balance can cause guard cell dysfunction and inhibit transpiration that limits nutrient absorption and photosynthesis (Armengaud et al., 2009). A decline in Ca, Mg, and B is possibly due to the limitation of nutrient intake, transport, or metabolism induced by HLB pathogen (Spann and Schumann, 2009). In HLB-affected trees, higher levels of N and Zn were observed, which may be due to plants physiological changes or the coexistence of other citrus diseases (Razi et al., 2011). It was reported that N, Mg, and Fe levels were lower in HLB-affected mandarin (*C. reticulata*) trees compared with healthy plants (Pustika et al., 2008).

## Conclusion

A sustainable strategy is critical for the U.S. citrus industry to recuperate infected plants by discovering environmental-friendly antimicrobials that can eliminate Las bacteria, improve canopy, productivity, and fruit quality. This study revealed that PEN combined with surfactant was most effective in controlling Las bacterial titers in citrus trees in over a 2-year trial. In addition, PEN treatment significantly increased the fruit yield and fruit weight while PEN and OXY treatments significantly improved the fruit qualities in infected citrus trees. All the chemical treatments were effective in restoring tree canopy though none of these treatments did cure the infected trees. These findings indicate chemotherapy with an effective compound is useful as a component of integrated control for commercial citrus production in HLB endemic regions.

## Data Availability Statement

The raw data supporting the conclusions of this article will be made available by the authors, without undue reservation.

## Author Contributions

YD, CP, and MZ conceived and designed the experiments. MZ, XS, DZ, and RF performed the experiments. MZ, PK, JB, and RF analyzed the data. CP, RF, and YD contributed reagents, materials, and analysis tools. MZ, PK, RF, CP, and YD wrote and revised the manuscript. All authors contributed to the article and approved the submitted version.

## Conflict of Interest

The authors declare that the research was conducted in the absence of any commercial or financial relationships that could be construed as a potential conflict of interest.

## Footnotes

1 https://citrusrdf.org/wp-content/uploads/2012/10/Field-Trial-Evaluation-Methods-for-Growers_updated-3-11-16ss-v3.pdf
