# Supplementary material for: Field Evaluation of Chemotherapy on HLB-Affected Citrus Trees With Emphasis on Fruit Yield and Quality
Source: Front Plant Sci. 2021 Feb 22;12:611287. doi: 10.3389/fpls.2021.611287 (PMC7953902; doi:10.3389/fpls.2021.611287)
Supplement: Supplementary file 1 [file Table_1.docx]

**Supplemental table 1. Antimicrobial residues in grapefruit juice extracted from fruit harvested on 2/13/2017 and 9/15/2017^a^**

| **Antibiotic** | **Residual (ppb)** | | **Maximum residue level** |
| --- | --- | --- | --- |
|  | 02/13/2017 | 09/15/2017 |  |
| Penicillin G potassium ^z^ | 0 | 0 | 10 ppb (EU) |
| Carvacrol ^y^ | 0 | 0 | GRAS (FDA) |
| Oxytetracycline ^x^ | 95.71 ± 9.16 | - ^w^ | 100 ppb (JECFA) |
| Validoxylamine A ^v^ | - | - | Not available |
| Aliette ^u^ | 0 | 0 | 5 ppm (US Citrus) ^*^ |

^z^: HPLC-MS method, detection limit 5 ppb

^y^: Headspace SPME GC-MS method, detection limit 2 ppb

^x^: ACCEL ELISA kit

^w^: No juice sample

^v^: Not determined due to analytical method not available.

^u^: LC-MS/MS method, detection limit 0.2 ppm

^*^: Reference: Ritenour, M. 2019. Maximum Residue Limits (MRLs) in part-per-million (ppm)

For Citrus - By Country. Available: https://irrec.ifas.ufl.edu/postharvest/pdfs/MRLs/Citrus_MRLs_12.13.19.pdf

**^a^ Method for residue assay**

Grapefruits were harvested from the trees treated with penicillin G potassium (control), carvacrol, oxytetracycline, or Aliette (aluminum tris(o-ethylphosphonate)) on 2/13/2017 and 9/15/2017. Each treatment contained three replicates of 15 fruits each. Fruits were manually washed using detergent (Fruit Cleaner 395; JBT FoodTech, Lakeland, FL), rinsed, and air dried. Fruits were then juiced using a hand-reamer juicer. Juice samples were stored at -20 °C until analysis. Penicillin G potassium was detected by HPLC-MS, Carvacrol by GC-MS, Oxytetracycline by ACCEL ELISA kit and Aliette by LC-MS/MS.
